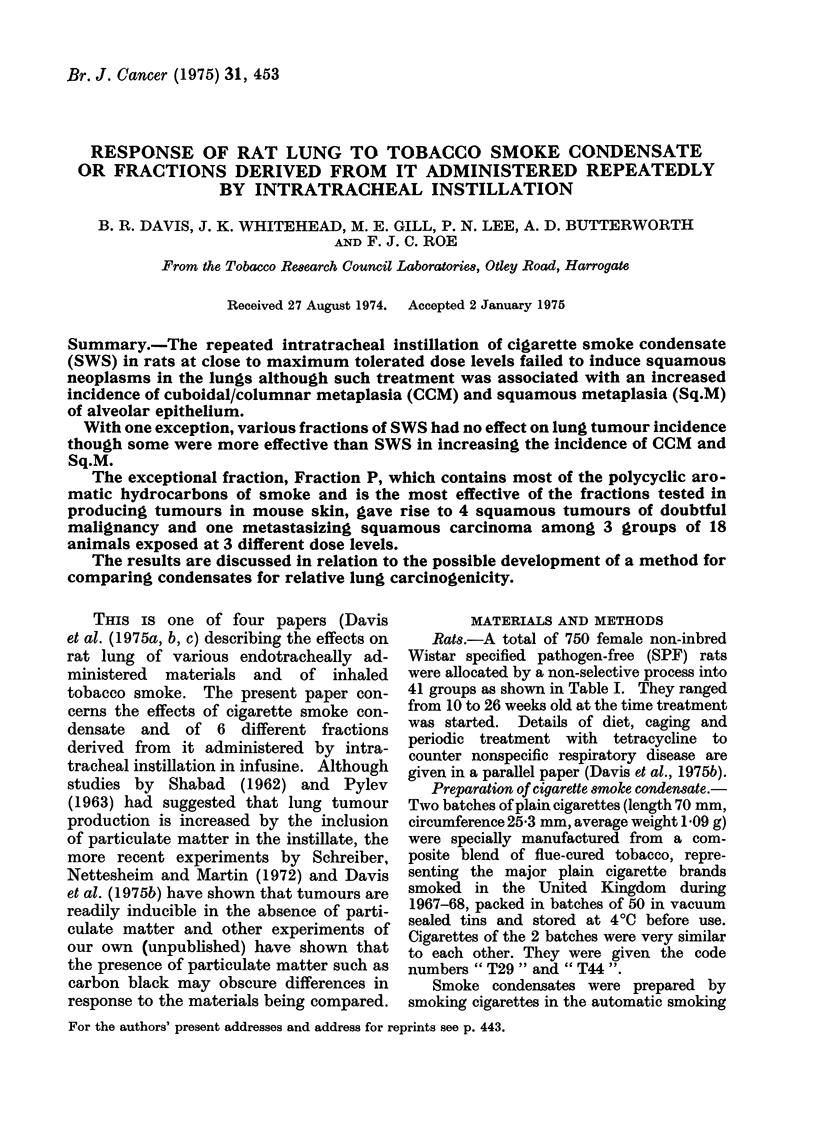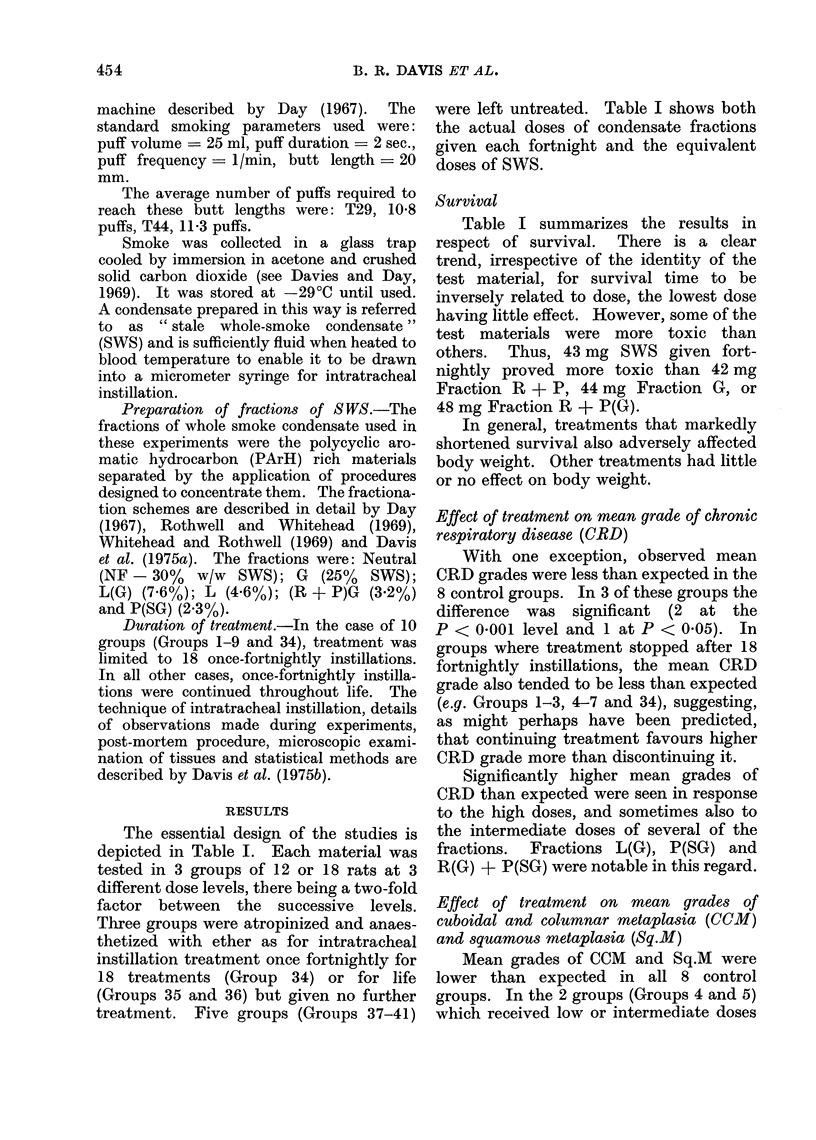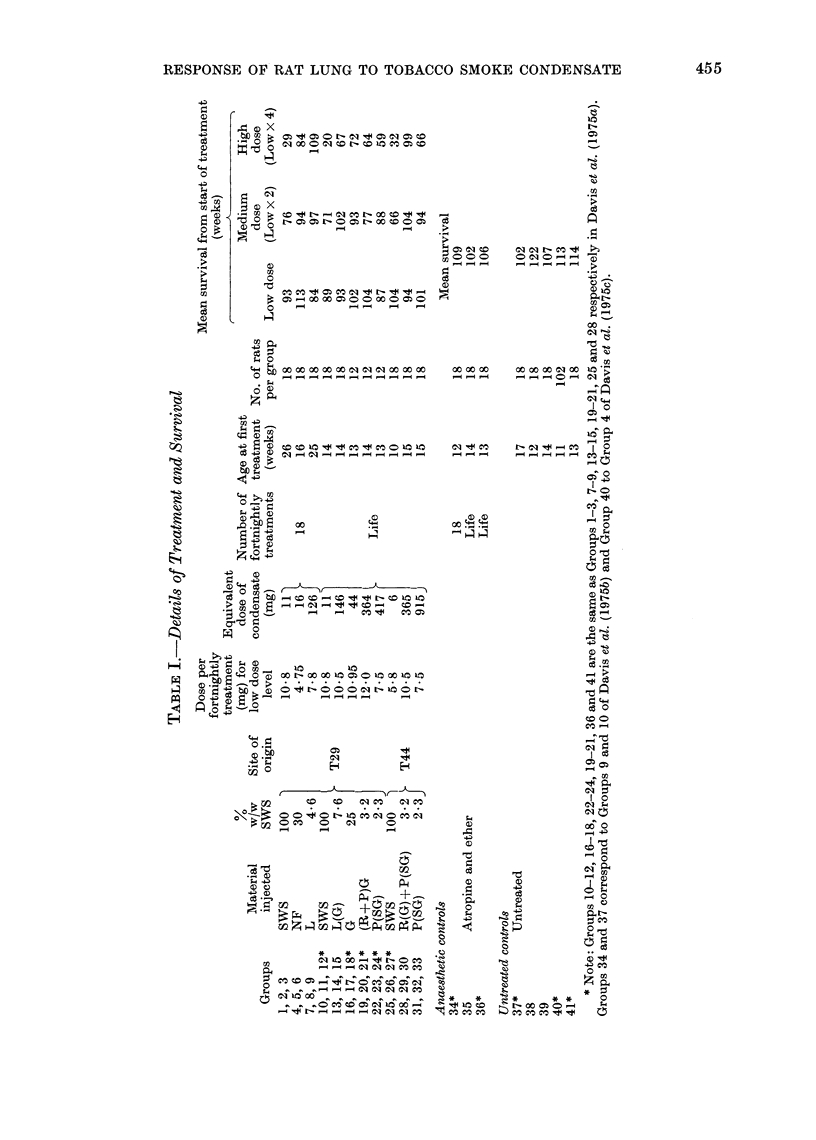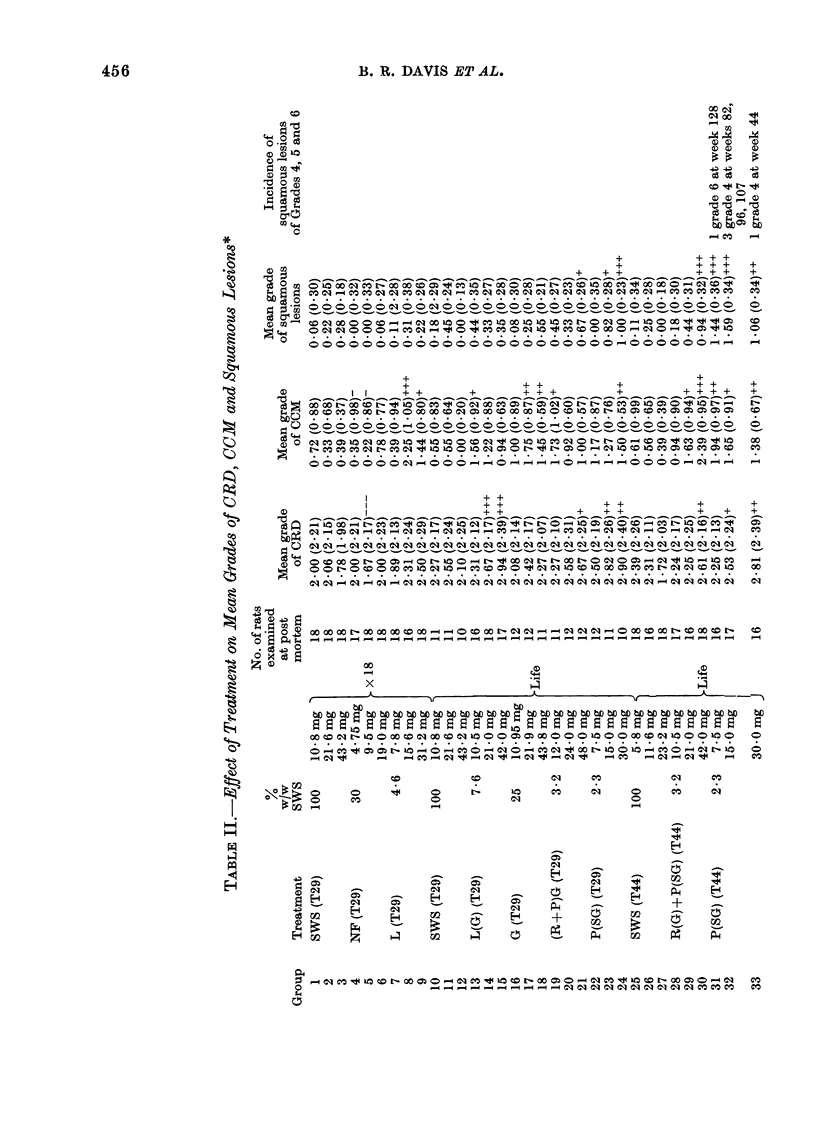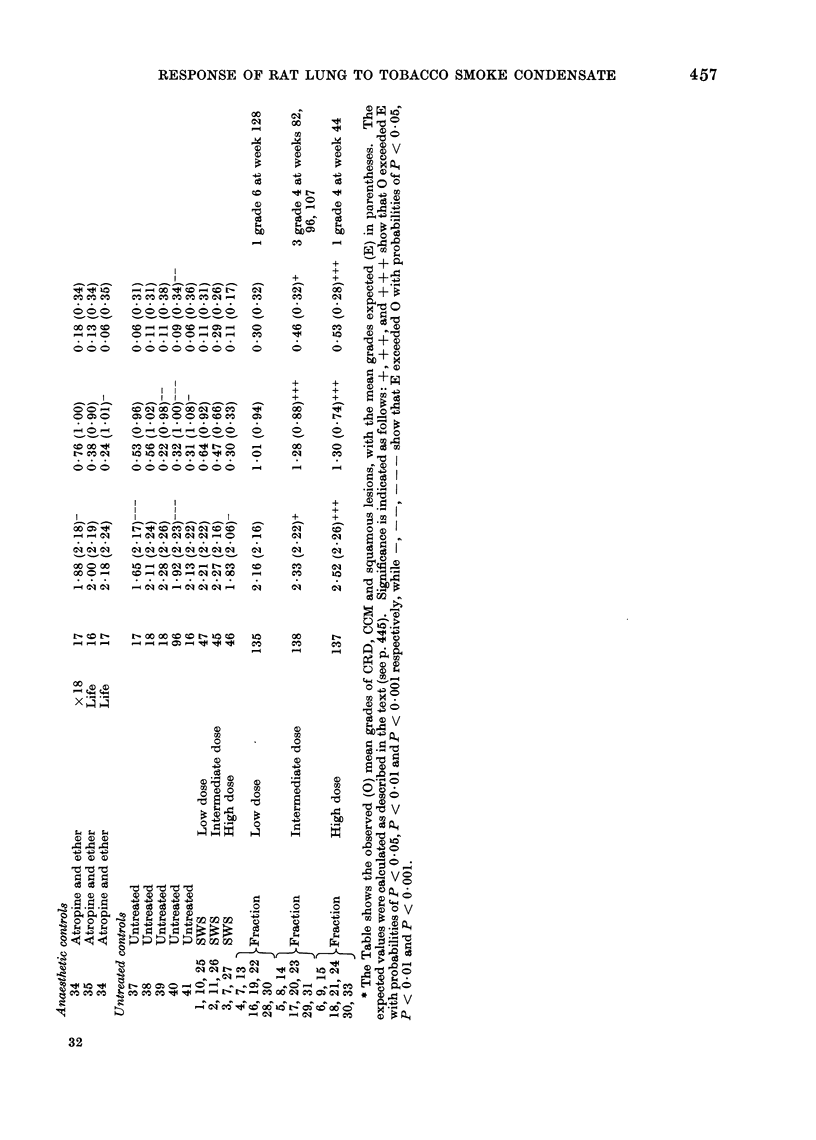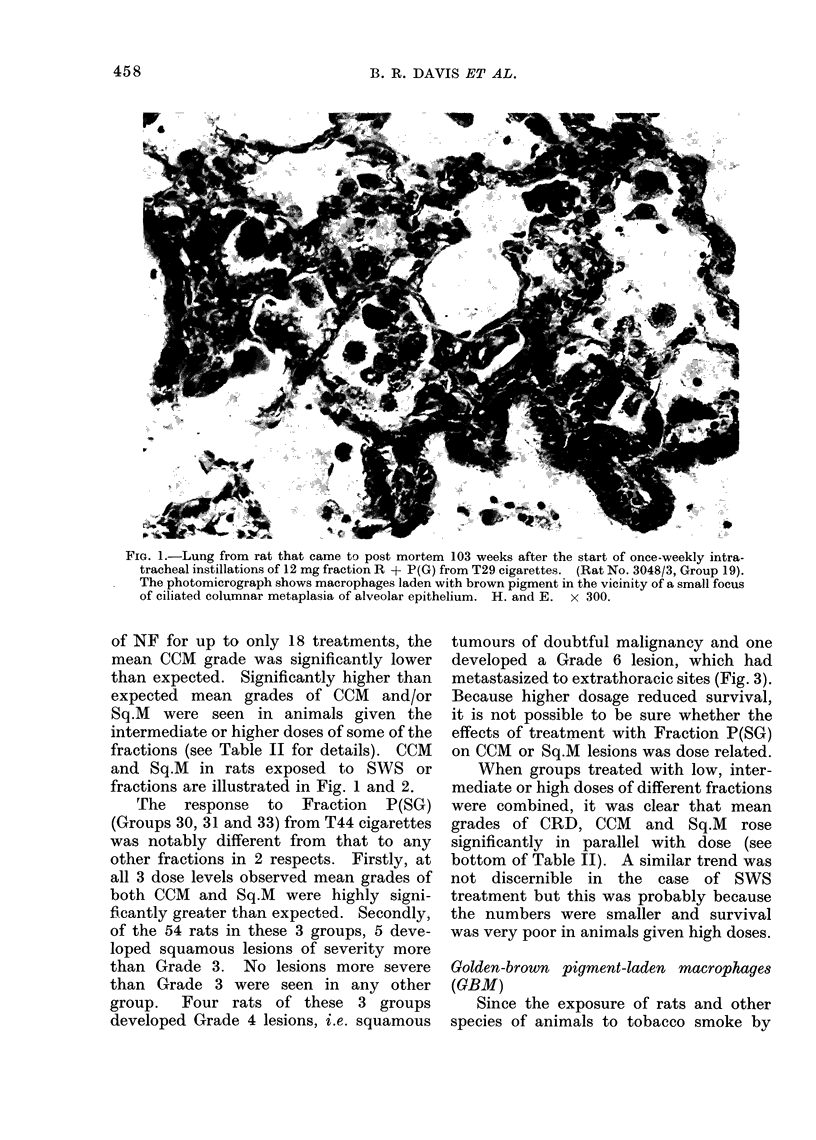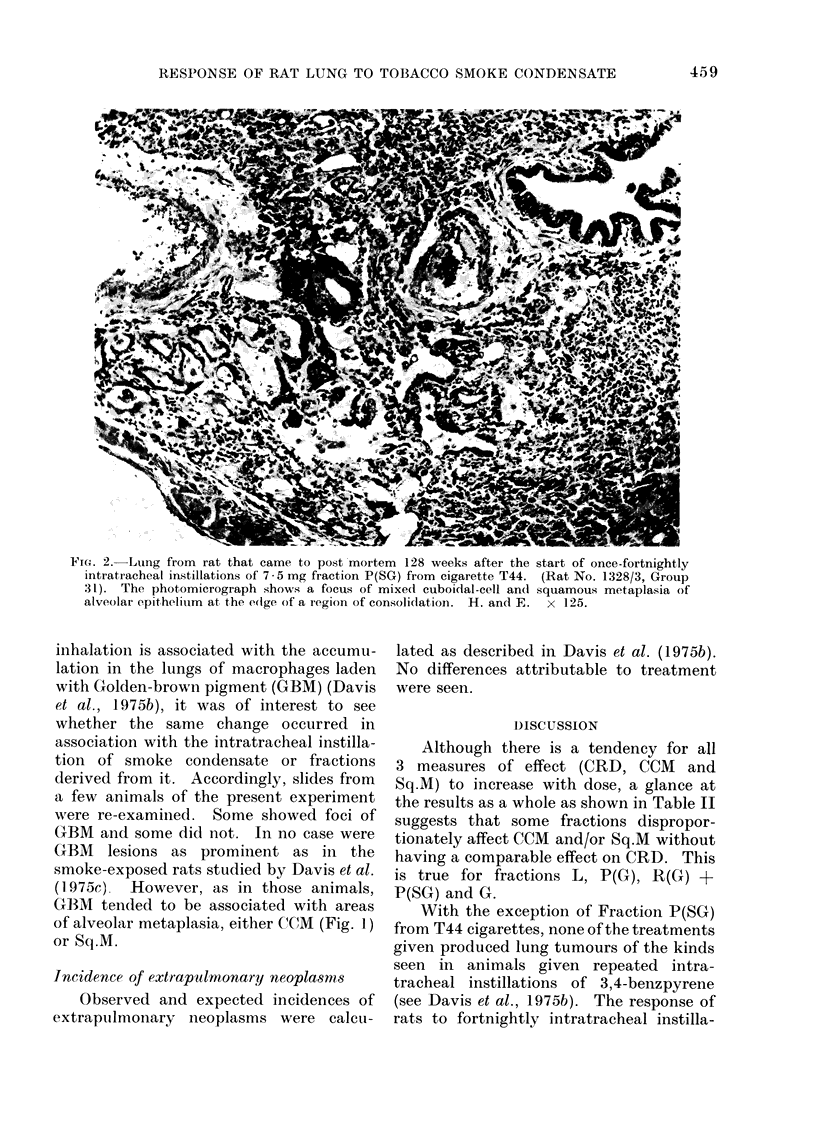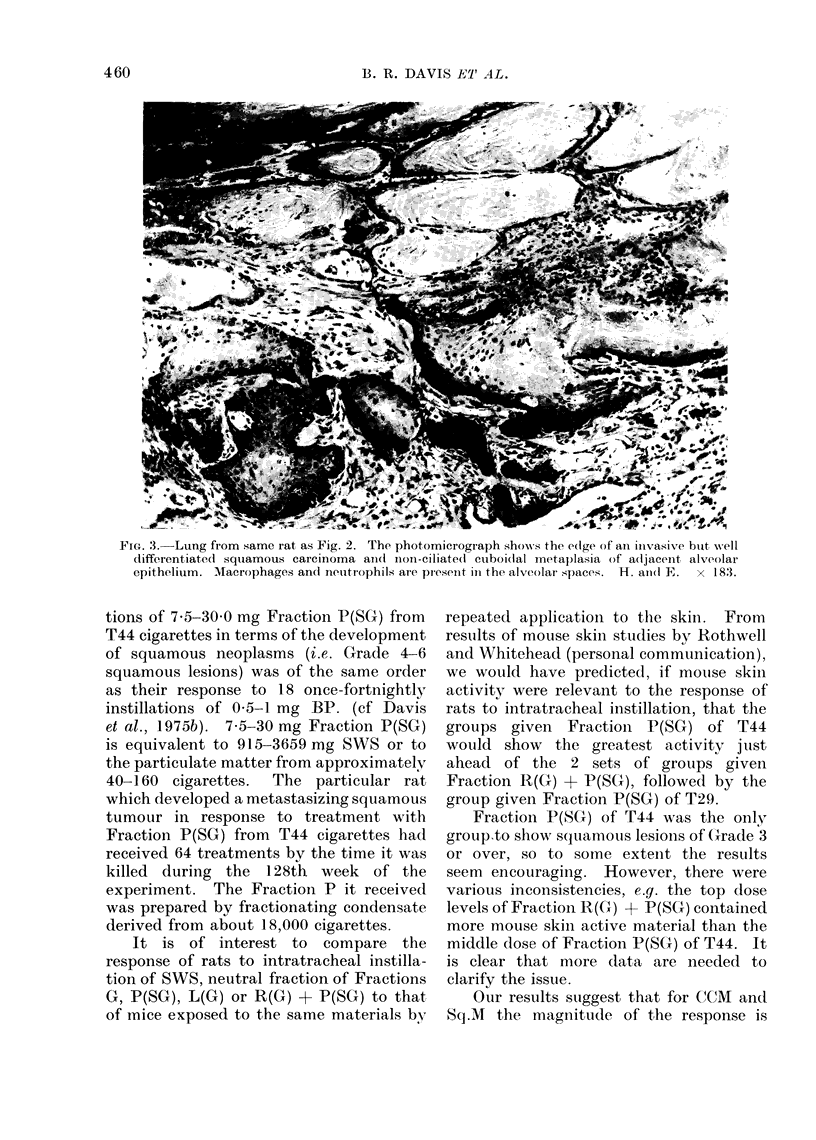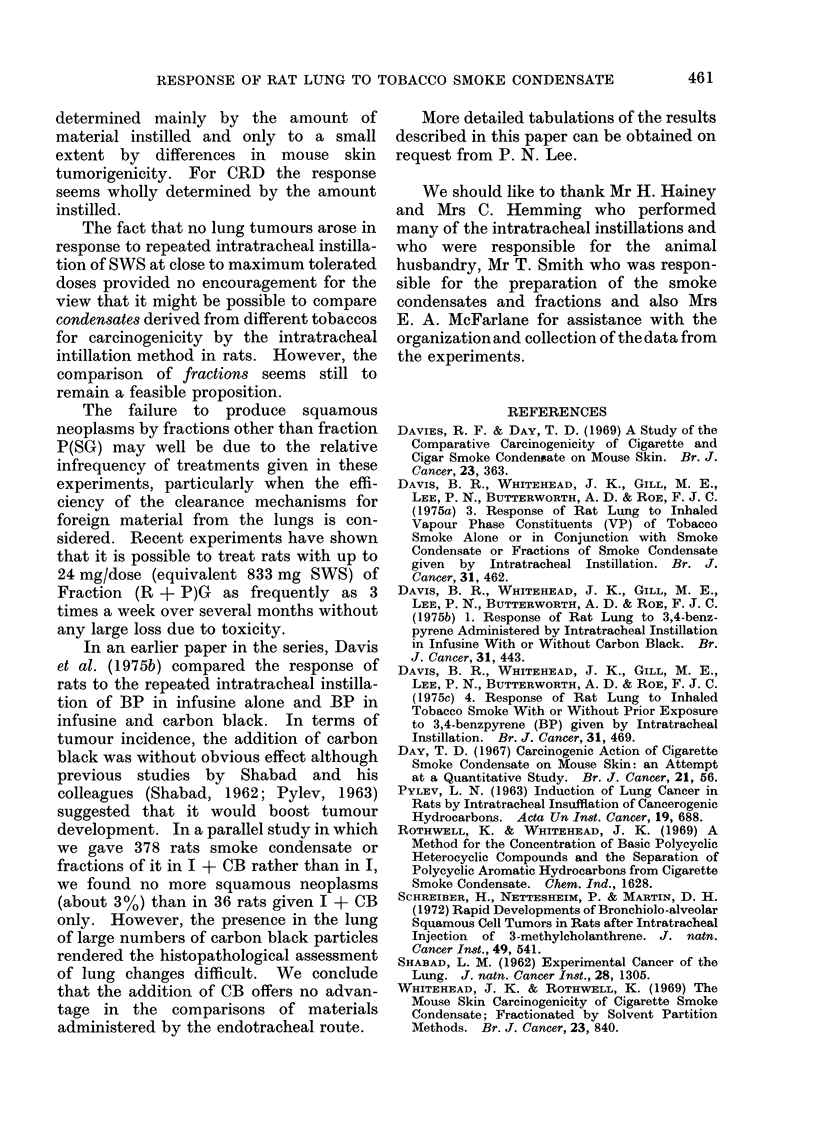# Response of rat lung to tobacco smoke condensate or fractions derived from it administered repeatedly by intratracheal instillation.

**DOI:** 10.1038/bjc.1975.85

**Published:** 1975-04

**Authors:** B. R. Davis, J. K. Whitehead, M. E. Gill, P. M. Lee, A. D. Butterworth, F. J. Roe

## Abstract

**Images:**


Br. J. Cancer (1975) 31, 453

### RESPONSE OF RAT LUNG TO TOBACCO SMOKE CONDENSATE OR FRACTIONS DERIVED FROM IT ADMINISTERED REPEATEDLY BY INTRATRACHEAL INSTILLATION

B. R. DAVIS, J. K. WHITEHEAD, M. E. GILL, P. N. LEE, A. D. BUTTERWORTH AND F. J. C. ROE

*From the Tobacco Research Council Laboratories, Otley Road, Harrogate*

Received 27 August 1974. Accepted 2 January 1975

**Summary.—The repeated intratracheal instillation of cigarette smoke condensate (SWS) in rats at close to maximum tolerated dose levels failed to induce squamous neoplasms in the lungs although such treatment was associated with an increased incidence of cuboidal/columnar metaplasia (CCM) and squamous metaplasia (Sq.M) of alveolar epithelium.**

**With one exception, various fractions of SWS had no effect on lung tumour incidence though some were more effective than SWS in increasing the incidence of CCM and Sq.M.**

**The exceptional fraction, Fraction P, which contains most of the polycyclic aromatic hydrocarbons of smoke and is the most effective of the fractions tested in producing tumours in mouse skin, gave rise to 4 squamous tumours of doubtful malignancy and one metastasizing squamous carcinoma among 3 groups of 18 animals exposed at 3 different dose levels.**

**The results are discussed in relation to the possible development of a method for comparing condensates for relative lung carcinogenicity.**

THIS IS one of four papers (Davis *et al.* (1975*a*, *b*, *c*) describing the effects on rat lung of various endotracheally administered materials and of inhaled tobacco smoke. The present paper concerns the effects of cigarette smoke condensate and of 6 different fractions derived from it administered by intratracheal instillation in infusine. Although studies by Shabad (1962) and Pylev (1963) had suggested that lung tumour production is increased by the inclusion of particulate matter in the instillate, the more recent experiments by Schreiber, Nettesheim and Martin (1972) and Davis *et al.* (1975*b*) have shown that tumours are readily inducible in the absence of particulate matter and other experiments of our own (unpublished) have shown that the presence of particulate matter such as carbon black may obscure differences in response to the materials being compared.

### MATERIALS AND METHODS

*Rats.*—A total of 750 female non-inbred Wistar specified pathogen-free (SPF) rats were allocated by a non-selective process into 41 groups as shown in Table I. They ranged from 10 to 26 weeks old at the time treatment was started. Details of diet, caging and periodic treatment with tetracycline to counter nonspecific respiratory disease are given in a parallel paper (Davis *et al.*, 1975*b*).

*Preparation of cigarette smoke condensate.*—Two batches of plain cigarettes (length 70 mm, circumference 25·3 mm, average weight 1·09 g) were specially manufactured from a composite blend of flue-cured tobacco, representing the major plain cigarette brands smoked in the United Kingdom during 1967–68, packed in batches of 50 in vacuum sealed tins and stored at 4°C before use. Cigarettes of the 2 batches were very similar to each other. They were given the code numbers " T29 " and " T44 ".

Smoke condensates were prepared by smoking cigarettes in the automatic smoking

For the authors' present addresses and address for reprints see p. 443.

machine described by Day (1967). The standard smoking parameters used were: puff volume = 25 ml, puff duration = 2 sec., puff frequency = 1/min, butt length = 20 mm.

The average number of puffs required to reach these butt lengths were: T29, 10·8 puffs, T44, 11·3 puffs.

Smoke was collected in a glass trap cooled by immersion in acetone and crushed solid carbon dioxide (see Davies and Day, 1969). It was stored at −29°C until used. A condensate prepared in this way is referred to as "stale whole-smoke condensate" (SWS) and is sufficiently fluid when heated to blood temperature to enable it to be drawn into a micrometer syringe for intratracheal instillation.

*Preparation of fractions of SWS.*—The fractions of whole smoke condensate used in these experiments were the polycyclic aromatic hydrocarbon (PArH) rich materials separated by the application of procedures designed to concentrate them. The fractionation schemes are described in detail by Day (1967), Rothwell and Whitehead (1969), Whitehead and Rothwell (1969) and Davis *et al.* (1975*a*). The fractions were: Neutral (NF — 30% w/w SWS); G (25% SWS); L(G) (7·6%); L (4·6%); (R + P)G (3·2%) and P(SG) (2·3%).

*Duration of treatment.*—In the case of 10 groups (Groups 1–9 and 34), treatment was limited to 18 once-fortnightly instillations. In all other cases, once-fortnightly instillations were continued throughout life. The technique of intratracheal instillation, details of observations made during experiments, post-mortem procedure, microscopic examination of tissues and statistical methods are described by Davis *et al.* (1975*b*).

### RESULTS

The essential design of the studies is depicted in Table I. Each material was tested in 3 groups of 12 or 18 rats at 3 different dose levels, there being a two-fold factor between the successive levels. Three groups were atropinized and anaesthetized with ether as for intratracheal instillation treatment once fortnightly for 18 treatments (Group 34) or for life (Groups 35 and 36) but given no further treatment. Five groups (Groups 37–41) were left untreated. Table I shows both the actual doses of condensate fractions given each fortnight and the equivalent doses of SWS.

### *Survival*

Table I summarizes the results in respect of survival. There is a clear trend, irrespective of the identity of the test material, for survival time to be inversely related to dose, the lowest dose having little effect. However, some of the test materials were more toxic than others. Thus, 43 mg SWS given fortnightly proved more toxic than 42 mg Fraction R + P, 44 mg Fraction G, or 48 mg Fraction R + P(G).

In general, treatments that markedly shortened survival also adversely affected body weight. Other treatments had little or no effect on body weight.

### *Effect of treatment on mean grade of chronic respiratory disease (CRD)*

With one exception, observed mean CRD grades were less than expected in the 8 control groups. In 3 of these groups the difference was significant (2 at the $P < 0·001$ level and 1 at $P < 0·05$). In groups where treatment stopped after 18 fortnightly instillations, the mean CRD grade also tended to be less than expected (*e.g.* Groups 1–3, 4–7 and 34), suggesting, as might perhaps have been predicted, that continuing treatment favours higher CRD grade more than discontinuing it.

Significantly higher mean grades of CRD than expected were seen in response to the high doses, and sometimes also to the intermediate doses of several of the fractions. Fractions L(G), P(SG) and R(G) + P(SG) were notable in this regard.

### *Effect of treatment on mean grades of cuboidal and columnar metaplasia (CCM) and squamous metaplasia (Sq.M)*

Mean grades of CCM and Sq.M were lower than expected in all 8 control groups. In the 2 groups (Groups 4 and 5) which received low or intermediate doses

FIG. 1.—Lung from rat that came to post mortem 103 weeks after the start of once-weekly intratracheal instillations of 12 mg fraction R + P(G) from T29 cigarettes. (Rat No. 3048/3, Group 19). The photomicrograph shows macrophages laden with brown pigment in the vicinity of a small focus of ciliated columnar metaplasia of alveolar epithelium. H. and E. × 300.

of NF for up to only 18 treatments, the mean CCM grade was significantly lower than expected. Significantly higher than expected mean grades of CCM and/or Sq.M were seen in animals given the intermediate or higher doses of some of the fractions (see Table II for details). CCM and Sq.M in rats exposed to SWS or fractions are illustrated in Fig. 1 and 2.

The response to Fraction P(SG) (Groups 30, 31 and 33) from T44 cigarettes was notably different from that to any other fractions in 2 respects. Firstly, at all 3 dose levels observed mean grades of both CCM and Sq.M were highly significantly greater than expected. Secondly, of the 54 rats in these 3 groups, 5 developed squamous lesions of severity more than Grade 3. No lesions more severe than Grade 3 were seen in any other group. Four rats of these 3 groups developed Grade 4 lesions, *i.e.* squamous tumours of doubtful malignancy and one developed a Grade 6 lesion, which had metastasized to extrathoracic sites (Fig. 3). Because higher dosage reduced survival, it is not possible to be sure whether the effects of treatment with Fraction P(SG) on CCM or Sq.M lesions was dose related.

When groups treated with low, intermediate or high doses of different fractions were combined, it was clear that mean grades of CRD, CCM and Sq.M rose significantly in parallel with dose (see bottom of Table II). A similar trend was not discernible in the case of SWS treatment but this was probably because the numbers were smaller and survival was very poor in animals given high doses.

*Golden-brown pigment-laden macrophages (GBM)*

Since the exposure of rats and other species of animals to tobacco smoke by

FIG. 2.—Lung from rat that came to post mortem 128 weeks after the start of once-fortnightly intratracheal instillations of 7·5 mg fraction P(SG) from cigarette T44. (Rat No. 1328/3, Group 31). The photomicrograph shows a focus of mixed cuboidal-cell and squamous metaplasia of alveolar epithelium at the edge of a region of consolidation. H. and E. × 125.

inhalation is associated with the accumulation in the lungs of macrophages laden with Golden-brown pigment (GBM) (Davis *et al.*, 1975*b*), it was of interest to see whether the same change occurred in association with the intratracheal instillation of smoke condensate or fractions derived from it. Accordingly, slides from a few animals of the present experiment were re-examined. Some showed foci of GBM and some did not. In no case were GBM lesions as prominent as in the smoke-exposed rats studied by Davis *et al.* (1975*c*). However, as in those animals, GBM tended to be associated with areas of alveolar metaplasia, either CCM (Fig. 1) or Sq.M.

*Incidence of extrapulmonary neoplasms*

Observed and expected incidences of extrapulmonary neoplasms were calculated as described in Davis *et al.* (1975*b*). No differences attributable to treatment were seen.

### DISCUSSION

Although there is a tendency for all 3 measures of effect (CRD, CCM and Sq.M) to increase with dose, a glance at the results as a whole as shown in Table II suggests that some fractions disproportionately affect CCM and/or Sq.M without having a comparable effect on CRD. This is true for fractions L, P(G), R(G) + P(SG) and G.

With the exception of Fraction P(SG) from T44 cigarettes, none of the treatments given produced lung tumours of the kinds seen in animals given repeated intratracheal instillations of 3,4-benzpyrene (see Davis *et al.*, 1975*b*). The response of rats to fortnightly intratracheal instilla-

FIG. 3.—Lung from same rat as Fig. 2. The photomicrograph shows the edge of an invasive but well differentiated squamous carcinoma and non-ciliated cuboidal metaplasia of adjacent alveolar epithelium. Macrophages and neutrophils are present in the alveolar spaces. H. and E. × 183.

tions of 7·5–30·0 mg Fraction P(SG) from T44 cigarettes in terms of the development of squamous neoplasms (*i.e.* Grade 4–6 squamous lesions) was of the same order as their response to 18 once-fortnightly instillations of 0·5–1 mg BP. (cf Davis *et al.*, 1975*b*). 7·5–30 mg Fraction P(SG) is equivalent to 915–3659 mg SWS or to the particulate matter from approximately 40–160 cigarettes. The particular rat which developed a metastasizing squamous tumour in response to treatment with Fraction P(SG) from T44 cigarettes had received 64 treatments by the time it was killed during the 128th week of the experiment. The Fraction P it received was prepared by fractionating condensate derived from about 18,000 cigarettes.

It is of interest to compare the response of rats to intratracheal instillation of SWS, neutral fraction of Fractions G, P(SG), L(G) or R(G) + P(SG) to that of mice exposed to the same materials by repeated application to the skin. From results of mouse skin studies by Rothwell and Whitehead (personal communication), we would have predicted, if mouse skin activity were relevant to the response of rats to intratracheal instillation, that the groups given Fraction P(SG) of T44 would show the greatest activity just ahead of the 2 sets of groups given Fraction R(G) + P(SG), followed by the group given Fraction P(SG) of T29.

Fraction P(SG) of T44 was the only group to show squamous lesions of Grade 3 or over, so to some extent the results seem encouraging. However, there were various inconsistencies, *e.g.* the top dose levels of Fraction R(G) + P(SG) contained more mouse skin active material than the middle dose of Fraction P(SG) of T44. It is clear that more data are needed to clarify the issue.

Our results suggest that for CCM and Sq.M the magnitude of the response is determined mainly by the amount of material instilled and only to a small extent by differences in mouse skin tumorigenicity. For CRD the response seems wholly determined by the amount instilled.

The fact that no lung tumours arose in response to repeated intratracheal instillation of SWS at close to maximum tolerated doses provided no encouragement for the view that it might be possible to compare *condensates* derived from different tobaccos for carcinogenicity by the intratracheal intillation method in rats. However, the comparison of *fractions* seems still to remain a feasible proposition.

The failure to produce squamous neoplasms by fractions other than fraction P(SG) may well be due to the relative infrequency of treatments given in these experiments, particularly when the efficiency of the clearance mechanisms for foreign material from the lungs is considered. Recent experiments have shown that it is possible to treat rats with up to 24 mg/dose (equivalent 833 mg SWS) of Fraction (R + P)G as frequently as 3 times a week over several months without any large loss due to toxicity.

In an earlier paper in the series, Davis *et al.* (1975*b*) compared the response of rats to the repeated intratracheal instillation of BP in infusine alone and BP in infusine and carbon black. In terms of tumour incidence, the addition of carbon black was without obvious effect although previous studies by Shabad and his colleagues (Shabad, 1962; Pylev, 1963) suggested that it would boost tumour development. In a parallel study in which we gave 378 rats smoke condensate or fractions of it in I + CB rather than in I, we found no more squamous neoplasms (about 3%) than in 36 rats given I + CB only. However, the presence in the lung of large numbers of carbon black particles rendered the histopathological assessment of lung changes difficult. We conclude that the addition of CB offers no advantage in the comparisons of materials administered by the endotracheal route.

More detailed tabulations of the results described in this paper can be obtained on request from P. N. Lee.

We should like to thank Mr H. Hainey and Mrs C. Hemming who performed many of the intratracheal instillations and who were responsible for the animal husbandry, Mr T. Smith who was responsible for the preparation of the smoke condensates and fractions and also Mrs E. A. McFarlane for assistance with the organization and collection of the data from the experiments.

### REFERENCES

DAVIES, R. F. & DAY, T. D. (1969) A Study of the Comparative Carcinogenicity of Cigarette and Cigar Smoke Condensate on Mouse Skin. *Br. J. Cancer*, **23**, 363.

DAVIS, B. R., WHITEHEAD, J. K., GILL, M. E., LEE, P. N., BUTTERWORTH, A. D. & ROE, F. J. C. (1975*a*) 3. Response of Rat Lung to Inhaled Vapour Phase Constituents (VP) of Tobacco Smoke Alone or in Conjunction with Smoke Condensate or Fractions of Smoke Condensate given by Intratracheal Instillation. *Br. J. Cancer*, **31**, 462.

DAVIS, B. R., WHITEHEAD, J. K., GILL, M. E., LEE, P. N., BUTTERWORTH, A. D. & ROE, F. J. C. (1975*b*) 1. Response of Rat Lung to 3,4-benzpyrene Administered by Intratracheal Instillation in Infusine With or Without Carbon Black. *Br. J. Cancer*, **31**, 443.

DAVIS, B. R., WHITEHEAD, J. K., GILL, M. E., LEE, P. N., BUTTERWORTH, A. D. & ROE, F. J. C. (1975*c*) 4. Response of Rat Lung to Inhaled Tobacco Smoke With or Without Prior Exposure to 3,4-benzpyrene (BP) given by Intratracheal Instillation. *Br. J. Cancer*, **31**, 469.

DAY, T. D. (1967) Carcinogenic Action of Cigarette Smoke Condensate on Mouse Skin: an Attempt at a Quantitative Study. *Br. J. Cancer*, **21**, 56.

PYLEV, L. N. (1963) Induction of Lung Cancer in Rats by Intratracheal Insufflation of Cancerogenic Hydrocarbons. *Acta Un Inst. Cancer*, **19**, 688.

ROTHWELL, K. & WHITEHEAD, J. K. (1969) A Method for the Concentration of Basic Polycyclic Heterocyclic Compounds and the Separation of Polycyclic Aromatic Hydrocarbons from Cigarette Smoke Condensate. *Chem. Ind.*, 1628.

SCHREIBER, H., NETTESHEIM, P. & MARTIN, D. H. (1972) Rapid Developments of Bronchiolo-alveolar Squamous Cell Tumors in Rats after Intratracheal Injection of 3-methylcholanthrene. *J. natn. Cancer Inst.*, **49**, 541.

SHABAD, L. M. (1962) Experimental Cancer of the Lung. *J. natn. Cancer Inst.*, **28**, 1305.

WHITEHEAD, J. K. & ROTHWELL, K. (1969) The Mouse Skin Carcinogenicity of Cigarette Smoke Condensate; Fractionated by Solvent Partition Methods. *Br. J. Cancer*, **23**, 840.